# Ultrasound super-resolution imaging for the assessment of renal allograft dysfunction: A pilot study

**DOI:** 10.1016/j.heliyon.2024.e36515

**Published:** 2024-08-17

**Authors:** Yugang Hu, Yumeng Lei, Meihui Yu, Yao Zhang, Xingyue Huang, Ge Zhang, Qing Deng

**Affiliations:** a Department of Ultrasound Imaging, Renmin Hospital of Wuhan University, Wuhan, 430061, China; b Department of Medical Ultrasound, China Resources & Wisco General Hospital, Wuhan University of Science and Technology, Wuhan, 430080, China

**Keywords:** Super-resolution imaging, Kidney transplantation, Renal allograft dysfunction, Ultrasound

## Abstract

**Background:**

The purpose of this study was to examine the feasibility and practical application of ultrasound (US) super-resolution imaging (SRI) in evaluating microvasculature and measuring renal allograft function.

**Methods:**

Sixteen consecutive patients who received kidney transplants were prospectively enrolled. The patients were assigned as: normal allograft function (n = 6), and allograft malfunction (n = 10). Localizing each potential contrast signal resulted in super-resolution images (SRI). SRI was utilized to assess micro-vessel density (MVD) and microvascular flow rate, whereas contrast-enhanced (CE) US images were statistically processed to get the time to peak (TTP) and peak intensity. Logistic regression was utilized to evaluate their relationship.

**Results:**

US SRI may be utilized effectively on allografts to show microvasculature with significantly higher resolution than typical color Doppler flow and CEUS pictures. In the multivariate analysis, MVD and TTP were significant US markers of renal allograft failure (p = 0.031 and p = 0.045). The combination of MVD and TTP produced an AUC of 0.783 (p < 0.05) for allograft dysfunction.

**Conclusions:**

SRI can accurately portray the microvasculature of renal allografts, while MVD and TTP are appropriate US markers for assessing renal allograft failure.

## 1 Introduction

Because of the large growth in the frequency of end-stage kidney failure (ESKD) and rising organ scarcity over the last few decades, the gap between organ availability and the number of persons urgently requiring kidney transplantation (KT) has increased dramatically [[1], [2], [3]]. Thus, early detection measures for kidney allograft malfunction may be crucial in assisting doctors and patients in making decisions. Renal capillaries, which transport oxygen and nutrients to the tubules, are essential for normal kidney function [4]. Several studies have revealed that renal capillary rarefaction is an important pathophysiological process that promotes renal fibrosis and is directly related to the progression of chronic kidney disease (CKD) to ESKD [5,6]. Furthermore, some studies have found a high association between microvascular density and the risk of acute kidney injury (AKI) [7,8]. However, imaging options for noninvasive assessment of kidney microvascular changes are still clinically limited, although several methods have been employed to access renal microvessels in animals [9,10].

Ultrasound (US) is the primary suggested imaging method for KT patients; however, US gives little information about the microcirculation of allograft kidneys. Super-resolution imaging (SRI) has been utilized to identify individual injected microbubbles and follow their motion with subwavelength resolution, allowing for micrometer-scale vascular and velocity maps these years [11,12]. In our earlier researches, we employed microvascular density (MVD) and microvascular flow rate (MFR) to differentiate between benign and malignant thyroid or breast nodules [13,14]. US SRI can detect renal microvessels with a resolution of 32 μm in a unilateral ischemia-reperfusion damage mouse model. Additionally, there is a link between renal fibrosis and lower MVD as measured by SRI [15]. However, no study has been regulated on the use of US SRI or comparable methods in KT patients [16], even though US SRI may provide additional information about microvessels in the transplanted kidney. Hence, in this study, we explored the feasibility and therapeutic value of US SRI for KT patients.

## 2 Materials and methods

### 2.1 Participants

This study included adult KT patients who underwent their first KT at Renmin Hospital of Wuhan University between April and May 2023. We only included patients with: age >18 years, KT in more than 3 months, high-quality analysis images, and frequent follow-up at our hospital (i.e., patients could visit our hospital semimonthly within 6 months after transplantation, monthly within 1 year after transplantation, at least bimonthly within 5 years after transplantation, or quarterly after 5 years). Patients with contraindications to ultrasonography contrast agents, two or more kidney transplants, transplant kidney artery stenosis, hydronephrosis, perirenal fluid collection, mental problems image capture incompatibility, or inadequate clinical data were excluded. Furthermore, we excluded patients who experienced acute allograft malfunction during the first 3 months after surgery. Finally, this exploratory study included 16 patients who underwent KT.

The study was approved by the Renmin Hospital of Wuhan University's Institutional Review Board (No. WDRY2022-K70) after all patients signed an informed consent form.

One week before the ultrasound test, demographic, clinical, and biochemical data were collected from hospital medical records.

### 2.2 Definition of allograft dysfunction

Patients with normal allograft function had consistent allograft function (serum creatinine 57–97 μmol/L in males, 41–73 μmol/L in women, and negative urine protein) for at least 6 months before admission [17]. The patients were separated as the normal allograft (n = 6) and allograft dysfunction (n = 10).

The estimated glomerular filtration rate (eGFR) was determined using the CKD-EPI equation [18].

### 2.3 US image acquisition

Before gathering the materials for this study, a senior radiologist received standardized KT training and conducted all US exams. Due to the renal graft's superficial position in the iliac fossa, all patients underwent US imaging with a US platform (Resona 9s, Mindray Co. Ltd., China) and an L14-5WU linear array transducer. To avoid motion during the scan, the patients were instructed to maintain their breath briefly. All measurements were repeated three times and averaged.

The Doppler angle was maintained between 0° and 60°. In each patient, the peak systolic velocity and end-diastolic velocity of the main renal and renal interlobular arteries were determined manually. The resistive index (RI) was calculated using the integrated software. An image displaying the spectrum and the calculated RI was saved for each patient. The three measurement results were averaged.

### 2.4 SRI image acquisition

US SRI images were obtained using a CEUS imaging plane with the same area of interest (ROI) as the B-mode. Dual-mode pictures were utilized for tracking microbubble signals inside the kidney after injecting the contrast agent. SonoVue microbubbles (Bracco, Milan, Italy) were administered immediately for 0.5-mL bolus. After that, participants were instructed to maintain breaths for around 10 s. More than 1000 CEUS images were captured at a median frames’ frequency of 80 Hz. All data were stored in DICOM and audio-video interleave formats for additional manipulation.

### 2.5 CEUS image acquisition

For the typical CEUS, real-time dual-mode pictures were utilized for guiding the visual area and tracking microbubble signals after injection. The imaging was kept consistent to ensure that all images covered the same territory. A conventional clinical CEUS test was carried out, with the remaining 2 mL of the microbubble solution quickly. Exceptions for the frame rate and all settings were maintained from the prior SRI study. A qualitative study of contrast signals inside the kidney yielded many metrics, including TTP, PI, and AUC.

### 2.6 US SR image processing

The SR image manipulation algorithm was executed offline using MATLAB (MathWorks, Natick, MA, USA). Each image frame underwent singular value decomposition (SVD) to eliminate clutter and background signals. Following SVD processing, each picture frame underwent personalization. All localizations obtained from all images were summed to form the final SR image, as previously described [13,14].

### 2.7 Statistical analysis

Continuous parameters with normal distributions were presented as mean ± SD. Data that varied from the typical distribution were given as the median (interquartile range). The two variables were compared using either the independent-sample *t*-test or the Mann-Whitney *U* test. Logistic regression analysis was utilized to discover the US characteristics that influence kidney allograft function. The diagnostic performance was evaluated using receiver operating characteristic (ROC) curves.

## 3 Results

### 3.1 Baseline characteristics

Table 1 highlights all the data for participants involved in this study. Patients in the allograft dysfunction group had elevated levels of blood urea nitrogen and serum creatinine, along with lower eGFR values, compared to the normal allograft group. There were no significant variations in age, gender, or timing of transplantation between the two groups. As for US parameters, patients in the normal allograft group had higher PI, MVD, and MFR, but lower TTP and AUC (all p < 0.05) compared to those in the dysfunction group.

**Table 1:** Characteristics of all patients in this study.

Characteristics	Normal allograft group	Allograft dysfunction group	P value
N	6	10	–
Age, years old	39.0 ± 12.5	39.4 ± 9.5	0.943
Gender, male, n (%)	2 (33.3)	5 (50.0)	0.547
Time since transplant, months	14.7 ± 7.0	23.5 ± 9.2	0.092
Laboratory results			
BUN, mmol/L	8.8 ± 3.6	18.2 ± 8.6	0.025
Serum creatinine, μmol/L	84.7 ± 11.8	246.9 ± 90.6	0.006
eGFR, mL/min	70.4 ± 9.6	31.6 ± 10.5	<0.001
B-mode parameters			
Kidney length, cm	10.6 ± 1.2	10.8 ± 0.4	0.537
Cortical thickness, cm	1.4 ± 0.2	1.3 ± 0.2	0.278
CDFI parameters			
PSV, cm/s	84.5 ± 13.6	88.9 ± 13.0	0.530
RIm	0.70 ± 0.04	0.72 ± 0.06	0.541
RIi	0.61 ± 0.02	0.61 ± 0.03	0.964
CEUS parameters			
TTP, s	22.4 ± 2.2	26.5 ± 2.9	0.010
PI, dB	22.7 ± 2.8	17.3 ± 3.3	0.005
AUC, dBs	1895.5 ± 295.2	1699.7 ± 391.9	0.311
SRI parameters			
MVD, %	31.0 ± 4.4	14.6 ± 5.1	<0.001
MFR, mm/s	19.2 ± 0.9	13.8 ± 1.6	<0.001

BUN, blood urea nitrogen, eGFR, estimated-glomerular filtration rate, CDFI, color doppler flow imaging, PSV, renal main arteries' peak systolic velocity, RIm, renal main arteries' resistive index, RIi, interlobular arteries' resistive index, CEUS, contrast-enhanced ultrasonography, TTP, time to peak of renal cortex, PI, cortical peak intensity, AUC, area under the time-intensity curve of renal cortex, SRI, super-resolution imaging, MVD, micro-vessel density, MFR, super-resolved microvascular flow rate.

### 3.2 US images of allograft

**Fig. 1:**
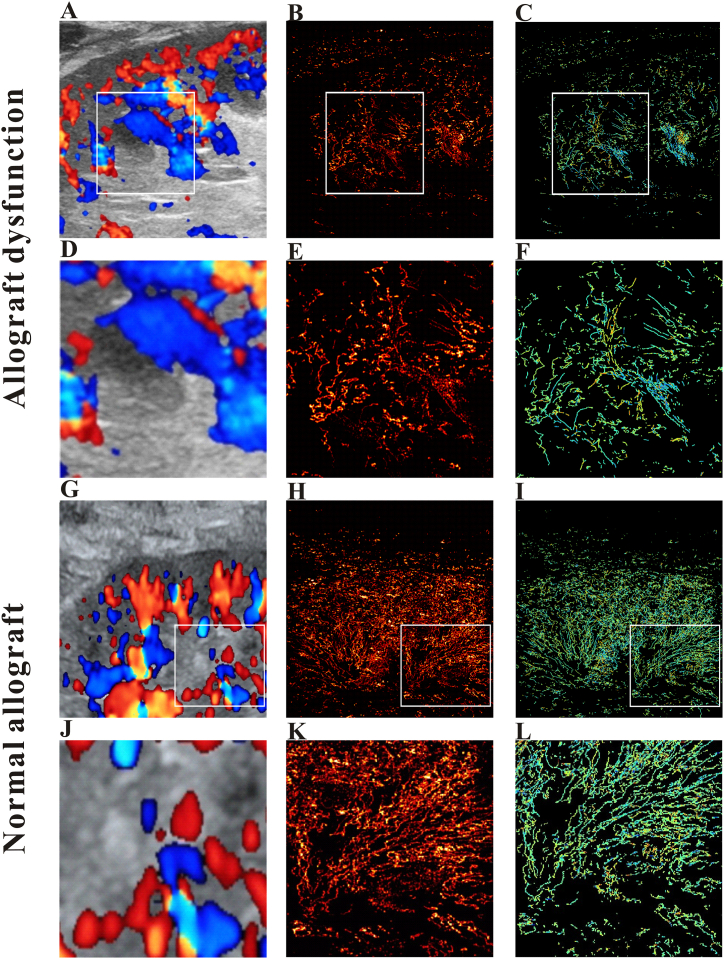
Ultrasound images of allograft dysfunction and normal allograft respectively. The color-doppler flow image **(A)**, super-resolution image **(B)**, super-resolved velocity image **(C)**, and the zoomed-in sections indicated as the white box of the color-doppler flow image **(D)**, super-resolution image **(E)**, super-resolved velocity image **(F)** for allograft dysfunction, respectively. The color-doppler flow image **(G)**, super-resolution image **(H)**, super-resolved velocity image **(I)**, and the zoomed-in sections indicated as the white box of the color-doppler flow image **(J)**, super-resolution image **(K)**, super-resolved velocity image **(L)** for normal allograft, respectively.

Fig. 1 depicts CDFI pictures (A, G), SRI images (B, H), SRVM images (C, I), and zoomed-in sections of the allograft's white box (D-F, J-L). Due to the low signal-to-noise ratio and angle dependency, CDFI images could only distinguish substantial arteries and fast blood flow (Fig. 1A–G). As a result, even though a patient had an allograft malfunction, there may not have been a substantial variation in blood vessel density. However, SRI can provide specific microvascular information that CDFI is unlikely to reveal. The SRI identified microvessels as tiny as a millimeter (Fig. 1E–K), but the SRVM recorded blood flow at much slower rates (Fig. 1F–L).

**Fig. 2:**
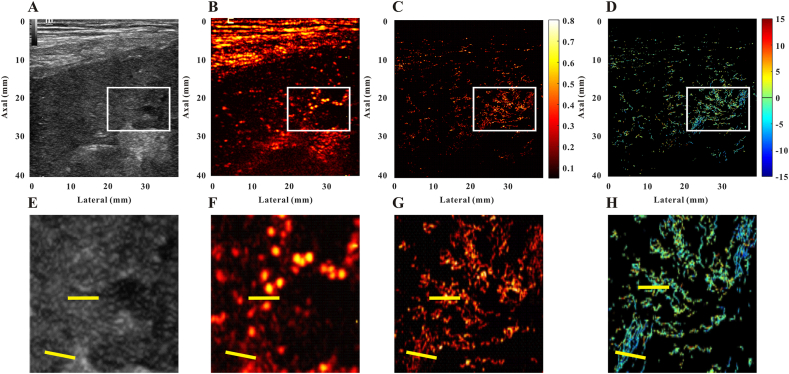
Representative images of an allograft dysfunction patient. B-mode image **(A)**, contrast-enhanced ultrasound image **(B)**, super-resolution image **(C)**, super-resolved velocity image **(D)**, and the zoomed-in regions indicated as the white box of the B-mode image **(E)**, contrast-enhanced ultrasound image **(F)**, super-resolution image **(G)**, super-resolved velocity image **(H)**. Yellow lines highlight the resolution improvement among the images.

**Fig. 3:**
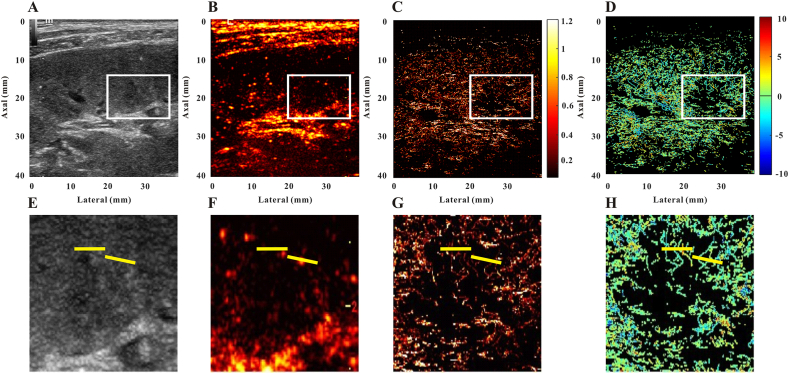
Representative images of a normal allograft patient. B-mode image **(A)**, contrast-enhanced ultrasound image **(B)**, super-resolution image **(C)**, super-resolved velocity image **(D)**, and the zoomed-in regions indicated as the red box of the B-mode image **(E)**, contrast-enhanced ultrasound image **(F)**, super-resolution image **(G)**, super-resolved velocity image **(H)**. Yellow lines highlight the resolution improvement among the images.

Fig. 2(A–H) and Fig. 3(A–H) show B-mode CEUS, SR, and SRVM images of a sample allograft malfunction and a normal allograft, respectively. The spatial resolution of conventional US limits its ability to reveal the microvascular architecture (Fig. 2, Fig. 3B–F). The SRI was able to surpass the ultrasonic diffraction limit and dramatically enhance the spatial resolution. After super-localization processing, at least two nearby micro-vessels and the tortuosity of micro-vessels in bulk were visible on the SR image (Fig. 2G, H and 3G, H) but not on CEUS. As a result, SRI can disclose the allograft's microvasculature in far better detail than CEUS. Moreover, the MVD and MFR values of the individual patient are described in Table 2.

**Table 2:** The SRI values and demographic data of all patients.

Patients	Age	Gender	Time (months)	MVD	MFR
Dysfunctional					
1	29	Male	18	10.97	14.20
2	40	Female	15	24.74	12.87
3	42	Male	21	16.72	13.74
4	45	Female	18	20.02	10.50
5	34	Male	21	10.95	13.76
6	54	Female	25	18.29	15.50
7	33	Female	16	11.37	15.96
8	51	Female	25	11.94	14.73
9	24	Male	40	10.27	12.54
10	42	Male	46	10.45	13.85
Normal					
1	46	Female	13	30.42	18.51
2	24	Male	11	31.66	19.88
3	29	Female	14	29.54	19.94
4	31	Female	6	36.35	19.27
5	51	Female	27	34.42	17.79
6	53	Male	17	23.55	19.78
SRI value (Mean ± SD)				20.73 ± 9.45	15.80 ± 3.01

SRI, super-resolution imaging, MVD, micro-vessel density, MFR, super-resolved microvascular flow rate, SD, standard deviation.

### 3.3 Correlation between US parameters and allograft dysfunction

TTP (OR, 1.65; 95 % CI, 1.02–2.86; p = 0.045) and MVD (OR, 0.65; 95 % CI, 0.29–0.93; p = 0.031) were independent risk factors for allograft dysfunction (Table 3).

**Table 3:** The association between US parameters and renal allograft function by logistic regression analysis.

	Univariate analysis		Multivatiate analysis	
	OR (95 % CI)	P	OR (95 % CI)	P
Kidney length	1.62 (0.38–6.89)	0.513		
Cortical thickness	0.11 (0.01–32.79)	0.268		
PSV	1.03 (0.95–1.12)	0.502		
RIm	67.81 (0.09–765.8)	0.513		
RIi	2.59 (0.07–1026.4)	0.961		
TTP	1.84 (1.04–3.25)	0.037	1.65 (1.02–2.86)	0.045
PI	0.21 (0.03–0.98)	0.046		
AUC	0.99 (0.98–1.01)	0.293		
MVD	0.52 (0.20–0.74)	0.010	0.65 (0.29–0.93)	0.031
MFR	1.04 (1.01–1.17)	0.035		

OR, odds ratio, 95%CI, 95 % confidence index, PSV, renal main arteries' peak systolic velocity, RIm, renal main arteries' resistive index, RIi, interlobular arteries' resistive index, TTP, time to peak of renal cortex, PI, cortical peak intensity, AUC, area under the time-intensity curve of renal cortex, MVD, micro-vessel density, MFR, super-resolved microvascular flow rate.

**Fig. 4:**
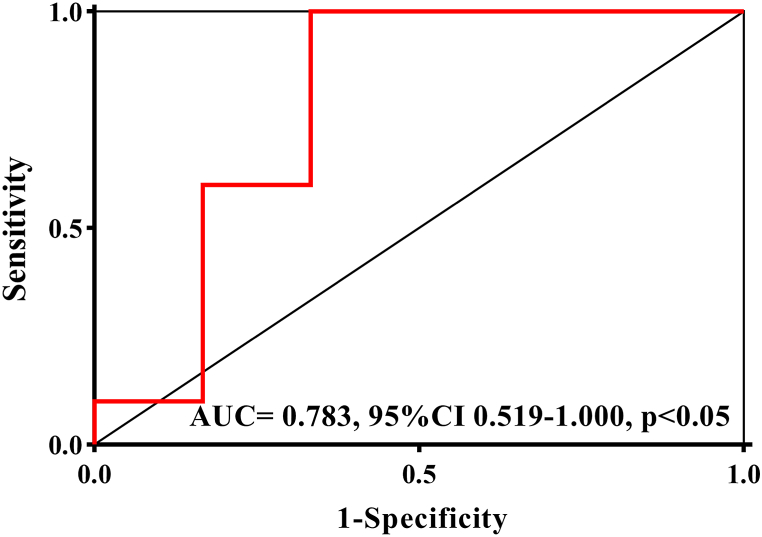
Graph shows the receiver operating characteristic curve of the combination of micro-vessel density (MVD) and time to peak (TTP) for allograft dysfunction.

ROC analysis revealed that both TTP and MVD exhibited general predictive accuracy for allograft dysfunction, with AUCs of 0.617 (95 % CI, 0.334–0.899) and 0.685 (95 % CI, 0.494–0.973). Using TTP and MVD to predict allograft dysfunction resulted in an AUC of 0.783 (95 % CI, 0.519–1.000) with a cutoff value of 0.947, a sensitivity of 90.0 %, and a specificity of 76.7 % (Fig. 4, p < 0.05).

## 4 Discussion

In this study, we used the US SRI method to investigate its clinical feasibility and diagnostic utility for allograft dysfunction, compared with CEUS and CDFI. We discovered that the US SRI could be used as a one-stop evaluation tool for monitoring renal microcirculation. Furthermore, US SRI could show microvessels from allografts at a substantially greater resolution than those of traditional CDF and CEUS images. More significantly, the combination of MVD and TTP, as quantitative metrics derived from US SRI and CEUS, exhibited a reasonably strong diagnostic value for allograft dysfunction.

The most commonly used technique of imaging for assessing both normal and transplanted kidneys is US. However, physicians and patients cannot obtain sufficient information on the structural and function claims from quantitative echogenicity in the US; consequently, better diagnosis of allograft malfunction after KT is critical in inpatient care. Fortunately, several methods have been applied to overcome these deficiencies in the past decades. Among them, US elastography may be the most prevalent tool for assessing allograft functioning. Sorana et al. prospectively performed shear-wave elastography (SWE) on 63 kT patients and found that cortical tissue stiffness was considerably greater in patients with allograft malfunction than in those with stable function [19]. Other investigations yielded similar findings [20]. Previous research has found some intriguing, if not contradicting, findings. Stephan et colleagues. conducted a prospective investigation of 20 kT patients who had SWE and magnetic resonance elastography (MRE), concluding that SWE was more sensitive to allograft failure and had greater picture quality than MRE [21]. In contrast, Paul et al. demonstrated that MRE could serve as a great predictor of graft loss/relapse, whereas SWE did not correlate with allograft dysfunction or other renal outcomes, in a prospective study of 27 kT patients [22]. These discrepancies in previous studies might be partly explained by their inclusion of small samples obtained from a single center, which inevitably results in selection bias.

CEUS may be another active imaging modality for allograft dysfunction [[23], [24], [25]]. Recently, Yang et al. explored the ability of conventional US and CEUS to assess the function and prognosis of 78 kT patients and found that CEUS parameters were significantly associated with allograft function and eGFR and could serve as an accurate predictor of the prognosis of KT patients [26]. Our study added evidence that CEUS parameters might have diagnostic value for allograft dysfunction, and we found that TTP, a quantitative parameter obtained from CEUS, was significantly associated with allograft dysfunction. Moreover, except for allograft dysfunction, CEUS was also found to have the most helpful diagnostic value for subclinical allograft rejection in KT patients without allograft dysfunction, among other noninvasive parameters of US [27]. In addition to the aforementioned methods, few studies have investigated the relationship between the US parameters and allograft function. Therefore, a convenient reliable, and relatively accurate imaging method is urgently needed for clinicians to manage patients undergoing KT promptly.

In the last few decades, because of the numerous advancements in US equipment, US SRI, which is used to pinpoint the position of US contrast materials within arteries for the nonsurgical exhibit of tiny blood vessels, has been extensively researched for its detection and predictive value for various diseases. Our team has established its diagnostic utility in identifying malignant and benign thyroid nodules and breast tumors [13,14]. In rats, US SRI may offer a noninvasive evaluation of renal microvasculature alterations in acute renal ischemia [28] and diabetic kidney disease [29].Chen et al. used SRI to quantify renal vasculature alterations during AKI-to-CKD development in mice and observed that SRI could detect renal microvessels with a resolution of 32 μm. More importantly, they also demonstrated that the vessel density estimated by SRI was significantly correlated with the density determined by histology (R^2^ = 0.77, p < 0.001), indicating that SRI could accurately assess renal microvascular density in mice [15]. Moreover, a recent study also explored ultrasound localization microscopy (ULM) in seven KT patients and found that it could identify thinner vessels (diameters, 0.3 ± 0.2 mm), compared with other Doppler techniques. They also observed a tendency in velocity as the microvessels moved away from the kidney capsule [16]; however, they did not provide quantitative parameters for KT patients, especially those with allograft dysfunction. In this study, we prospectively performed US SRI examination for 16 kT patients and demonstrated that US SRI could acquire more information on renal vessels, compared with CDFI and CEUS. Furthermore, MVD, a quantitative parameter from US SRI, was significantly associated with allograft dysfunction. More importantly, the combination of MVD and TTP had a relatively good diagnostic value for allograft dysfunction.

There are also some disadvantages in the current investigation. First, a bigger sample size may be necessary to establish a clearer conclusion on US SRI in renal allograft dysfunction. However, data collection with SRI is problematic, as is the case with all SRI experiments on humans. Second, SRI production is time-consuming procedure, which may limit its clinical usage. Furthermore, because KT patients are often hesitant to undergo kidney biopsy, the diagnosis of allograft dysfunction has almost exclusively relied on laboratory data, and subclinical acute allograft failure may have gone undetected. Finally, we only observed baseline US characteristics; additional US tests throughout follow-up might help us better understand the function of US in prognosis prediction.

## 5 Conclusions

Compared to CDFI and CEUS, SRI can increase microvessel clearance in renal allografts. US SRI combined with CEUS can be a useful innocuous formation of image method for distinguishing between normal and defective allografts. Hence, we advocate employing US SRI as a innocuous approach in conjunction with existing laboratory and Doppler-based measures. This is expected to improve clinical decision-making when selecting patients for allograft biopsy.

## Data availability statement

All data used in this study are available from the corresponding author on a reasonable request.

## Funding

Not applicable.

## CRediT authorship contribution statement

**Yugang Hu:** Writing – review & editing, Writing – original draft, Validation, Software, Methodology, Investigation, Formal analysis. **Yumeng Lei:** Writing – review & editing, Writing – original draft, Software, Resources, Methodology, Investigation, Formal analysis, Data curation. **Meihui Yu:** Writing – review & editing, Writing – original draft, Software, Methodology, Investigation, Formal analysis, Data curation. **Yao Zhang:** Writing – review & editing, Writing – original draft, Investigation, Formal analysis, Data curation, Conceptualization. **Xingyue Huang:** Writing – review & editing, Writing – original draft, Methodology, Investigation, Data curation, Conceptualization. **Ge Zhang:** Writing – review & editing, Writing – original draft, Supervision, Software, Project administration, Methodology, Formal analysis, Data curation, Conceptualization. **Qing Deng:** Writing – review & editing, Writing – original draft, Supervision, Software, Resources, Project administration, Methodology, Investigation, Data curation.

## Declaration of competing interest

The authors declare that they have no known competing financial interests or personal relationships that could have appeared to influence the work reported in this paper.
